# Mutagenicity and safety pharmacology of a standardized antidiabetic polyherbal formulation

**DOI:** 10.1038/s41598-022-11243-3

**Published:** 2022-05-03

**Authors:** Fadzilah Adibah Abdul Majid, Anis Fadhlina, Hassan Fahmi Ismail, Siti Nurazwa Zainol, Archan Kumar Mamillapalli, Vijayabalaji Venkatesan, Rajesh Eswarappa, Renuka Pillai

**Affiliations:** 1 Institute of Marine Biotechnology, Universiti Malaysia Terengganu, 21030 Kuala Nerus, Terengganu Malaysia; 2 Institute of Food Security and Sustainable Agriculture, Universiti Malaysia Kelantan, Jeli campus, 17600 Jeli, Kelantan Malaysia; 3 Proliv Life Sciences Sdn Bhd, D-1-15, Residensi Bistaria, Jln Ulu Kelang, Taman Ukay Bistari, 68000 Ampang, Selangor Malaysia; 4 Aurigene Pharmaceutical Services Limited, Bollaram Road, Miyapur, Hyderabad, Telangana 500 049 India

**Keywords:** Drug safety, Pharmaceutics

## Abstract

Synacinn is a standardized polyherbal extract formulated for the treatment of diabetes mellitus and its complications. This study aims to assess the mutagenicity potential of Synacinn by Ames assay and in vivo bone marrow micronucleus (MN) test on Sprague Dawley rat. Human ether-a-go-go-related gene (hERG) assay and Functional Observation Battery (FOB) were done for the safety pharmacology tests. In the Ames assay, Dose Range Finding (DRF) study and mutagenicity assays (+/− S9) were carried out. For the MN test, a preliminary and definitive study were conducted. In-life observations and number of immature and mature erythrocytes in the bone marrow cells were recorded. The hERG assay was conducted to determine the inhibitory effect on hERG potassium channel current expressed in human embryonic kidney cells (HEK293). FOB tests were performed orally (250, 750, and 2000 mg/kg) on Sprague Dawley rats. Synacinn is non-mutagenic against all tested strains of *Salmonella typhimurium* and did not induce any clastogenicity in the rat bone marrow. Synacinn also did not produce any significant inhibition (*p* ≤ 0.05) on hERG potassium current. Synacinn did not cause any neurobehavioural changes in rats up to 2000 mg/kg. Thus, no mutagenicity, cardiotoxicity and neurotoxicity effects of Synacinn were observed in this study.

## Introduction

Over the past decade, herbal therapies are have become more popular and widely used for the treatment and prevention of various diseases because it is considered to be generally effective and safe. However, there are increasing concerns regarding their potential to produce adverse effects which may occur from incorrect raw materials during harvesting and inappropriate preparation of toxic ingredients as well as poor pharmaceutical quality control and overdosed administration^1^. In most countries, herbal products are commonly consumed as dietary supplements with no strict regulation on their quality control and safety. Various reports on adverse effects of herbal products have been associated not only with liver^2^ and kidney^3^ problems but also affecting the nervous system^4^, cardiovascular system^5^, mutagenicity^6^, and carcinogenicity^7^.

Mutagenicity may occur due to the point mutations which involve substitution, addition, or deletion of one or a few DNA base pairs while clastogenicity involves structural changes of chromosomes upon exposure to the genotoxic agents^8^. Study on the genotoxicity of herbal medicines is crucial as this data are required to support the safety of herbal products as well as one of the regulatory requirements in the registration of natural products with therapeutic claims^9^. Besides, several safety pharmacology tests have been developed and required to be completed prior to human exposure which specializing in detecting and investigating potential undesirable pharmacodynamic effects of new chemical entities (NCEs) on physiological functions concerning exposure in the therapeutic range and above. ICH S7A (Safety pharmacology studies for human pharmaceuticals) guideline aims to protect clinical trial participants (Phase I, II, and III) from accidental adverse effects of new chemical entities (NCEs) and minimize the undesirable pharmacodynamic effects during the drug development phases. The mandatory and comprehensive nonclinical safety pharmacology integrates in-silico, in vitro, and in vivo experimental approaches. Safety pharmacology detected undesirable pharmacodynamic events particularly on three primary organ systems (core battery systems) including the central nervous system (CNS), cardiovascular system, and respiratory system as well as implement supplementary tests on the gastrointestinal and renal system^10^.

Synacinn, a standardized polyherbal extract (*Andrographis paniculata, Curcuma xanthorrhiza, Cinnamomum zeylanicum, Eugenia polyantha* and *Orthosiphon stamineous*) has been recommended for diabetes mellitus treatment and testimonials claimed to reduce diabetes mellitus related signs including tiredness and high blood glucose level. Besides, multiple biological evidences were reported on Synacinn. The potential of herb-drug interaction of Synacinn and its biomarkers (rosmarinic acid, gallic acid, curcumin, catechin, and andrographolide) was evaluated through cytochrome P450 (CYP450) inhibition assay^11^. It was found that inhibitory activity of Synacinn to all tested CYP450 enzymes (CYP1A2, CYP2B6, CYP2C8, CYP2C9, CYP2C19, CYP2D6, CYP3A4, and CYP3A4) at 5 mg/mL was associated with curcumin. In another recent study on Synacinn^12^, preclinical toxicity and anti-hyperglycemic effects of Synacinn were conducted on streptozotocin (STZ)-induced type 1 diabetic rats. The study suggested that Synacinn was non-toxic to rats at a dose of 250 mg/kg and effectively acts as an anti-hyperglycemic agent at twice-daily 250 mg/kg dose in the STZ-induced diabetic rats.

In the present study, the mutagenicity potential of Synacinn was evaluated by in vitro Ames assay and in vivo Micronucleus (MN) bone marrow test. The safety pharmacodynamics test was also conducted focusing on the two ‘core battery subjects’; cardiovascular and central nervous system. For cardiovascular safety test, an in vitro patch-clamp technique was used to determine its inhibitory effect on the hERG potassium channel in HEK 293 cells. For neurobehavioural evaluation, stratified randomized Sprague Dawley rats were treated with Synacinn orally and the Functional Observation Battery (FOB) score was recorded according to the ICH S7A guideline (2000)^13^.

## Results

### Ames assay

**Figure 1:**
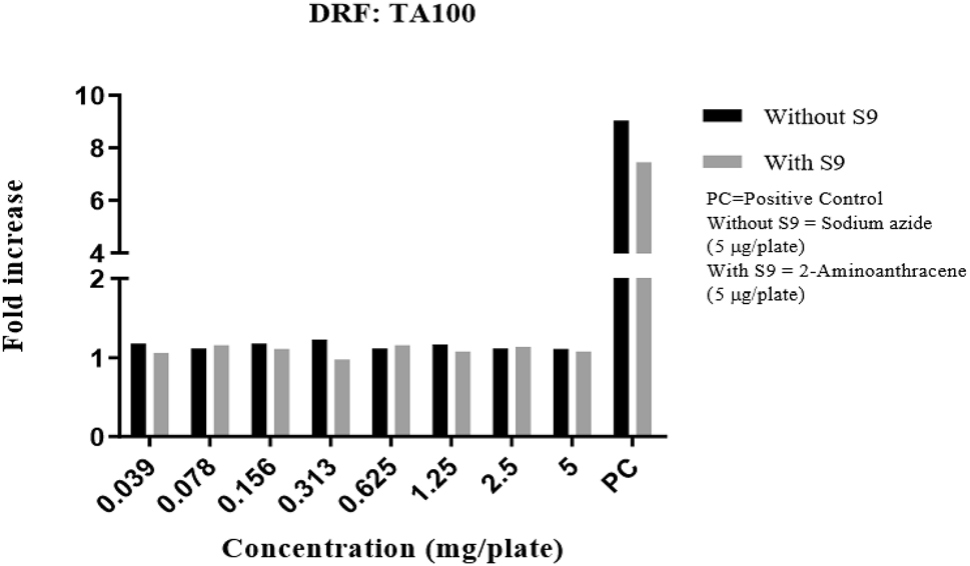
TA100: fold increase in revertant colony count (vs vehicle control): plate incorporation method (DRF).

**Figure 2:**
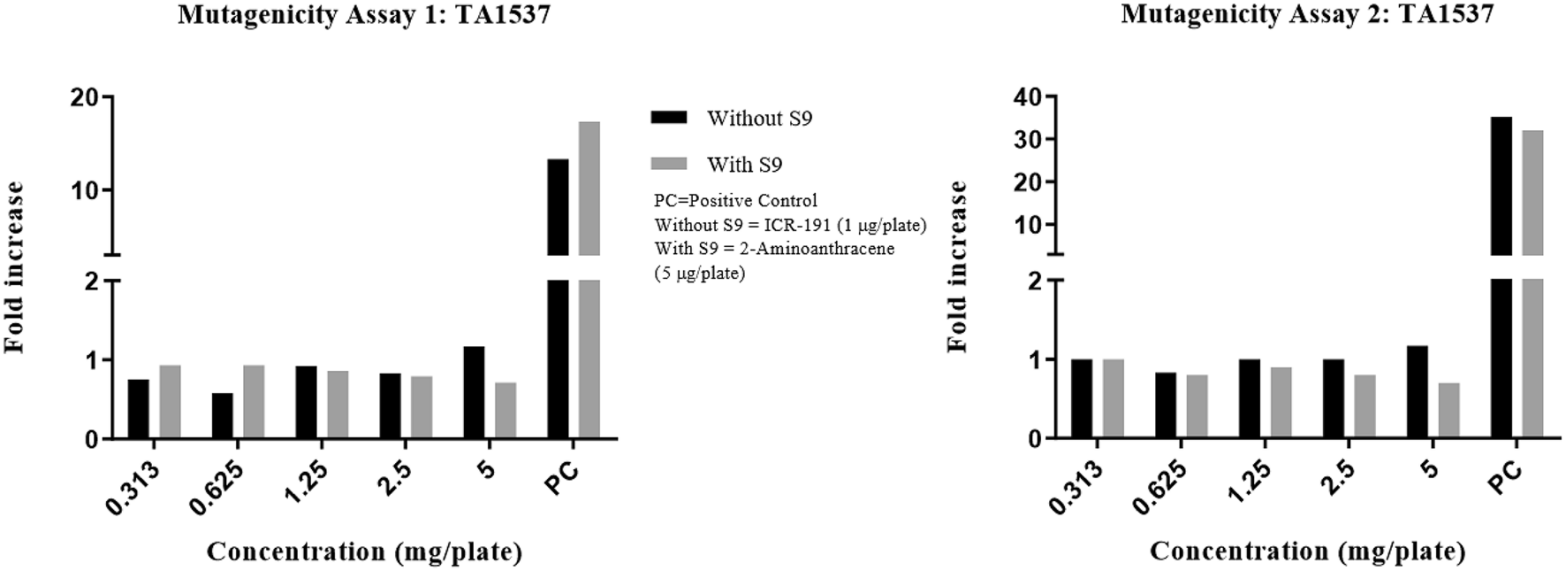
TA1537: fold increase in revertant colony count (vs vehicle control): mutagenicity assay 1 and 2.

**Figure 3:**
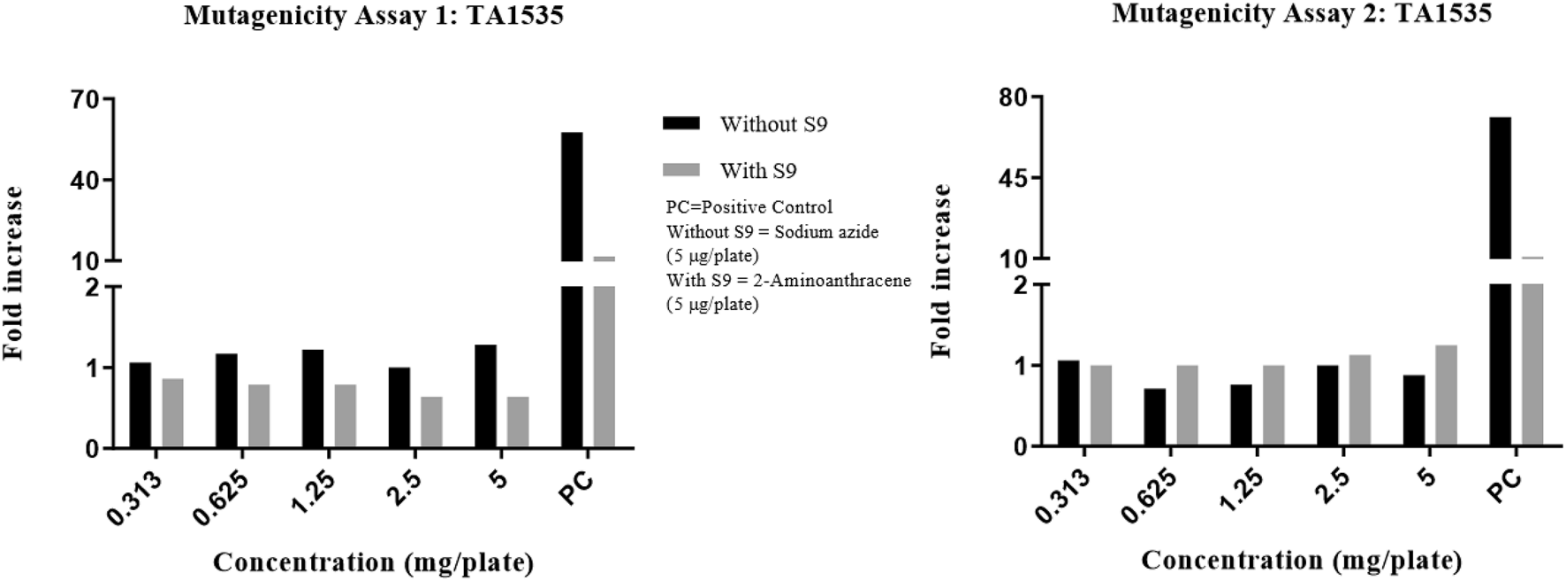
TA1535: fold increase in revertant colony count (vs vehicle control): mutagenicity assay 1 and 2.

**Figure 4:**
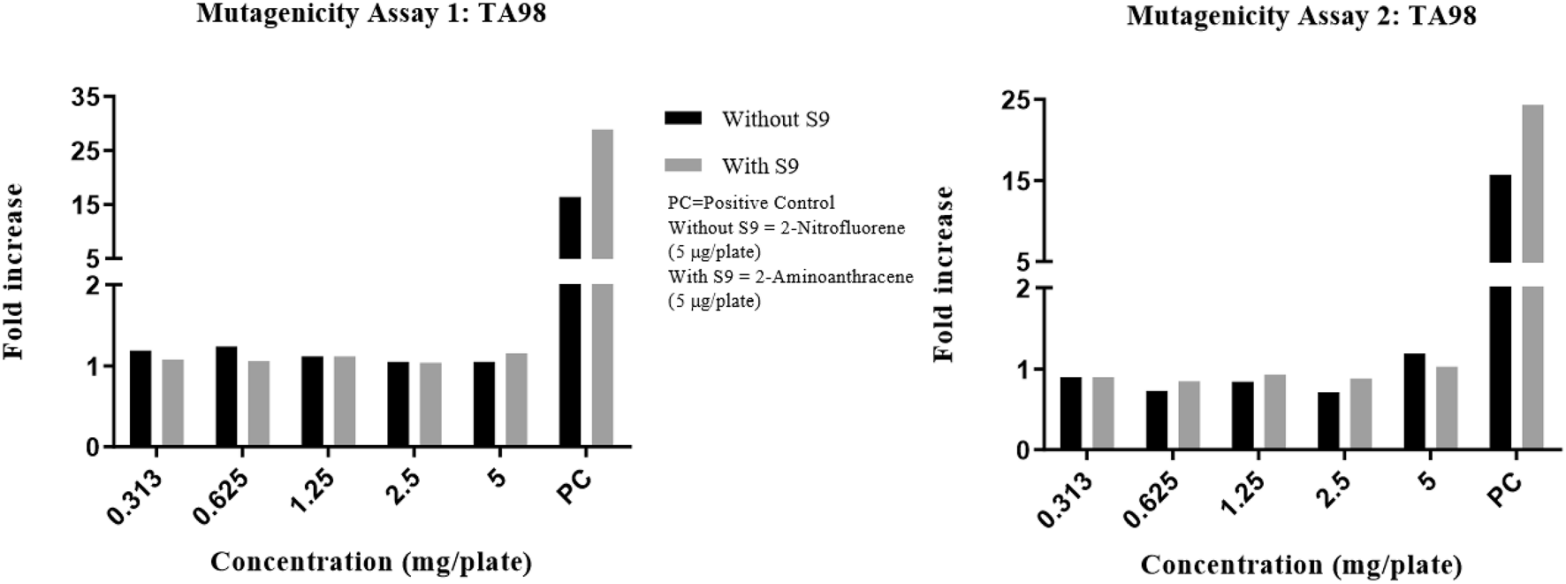
TA98: fold increase in revertant colony count (vs vehicle control): mutagenicity assay 1 and 2.

**Figure 5:**
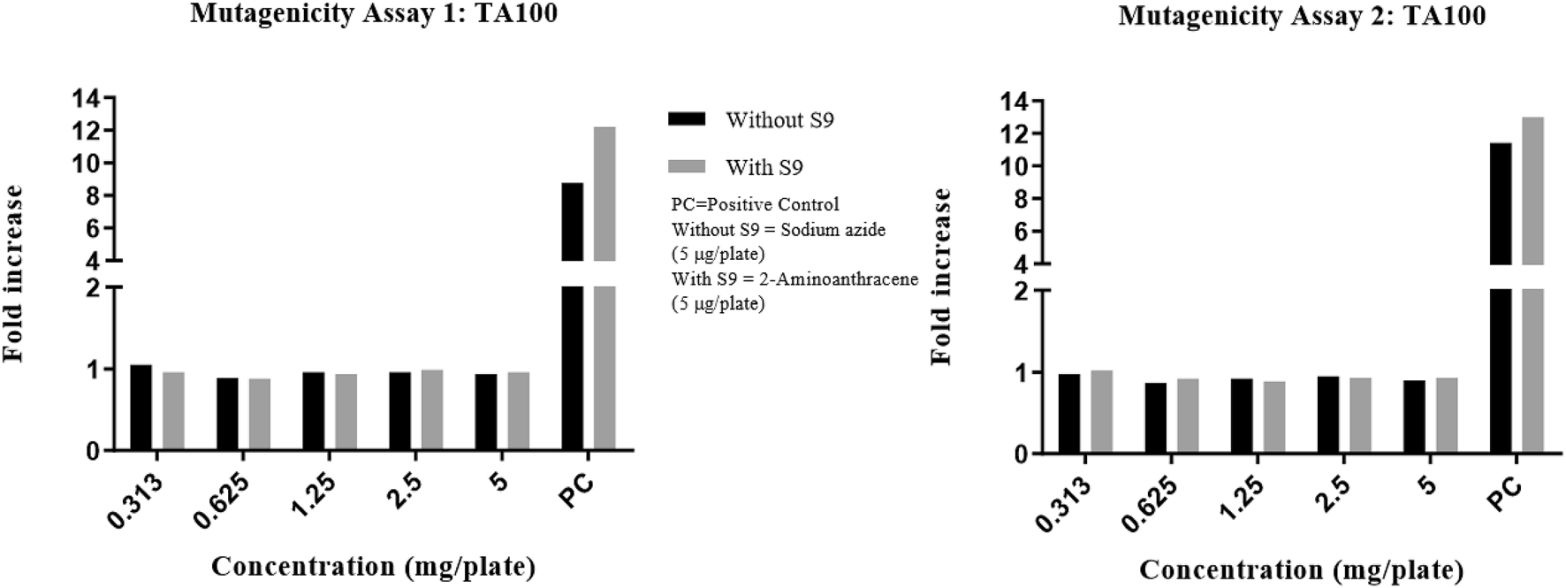
TA100: fold increase in revertant colony count (vs vehicle control): mutagenicity assay 1 and 2.

**Figure 6:**
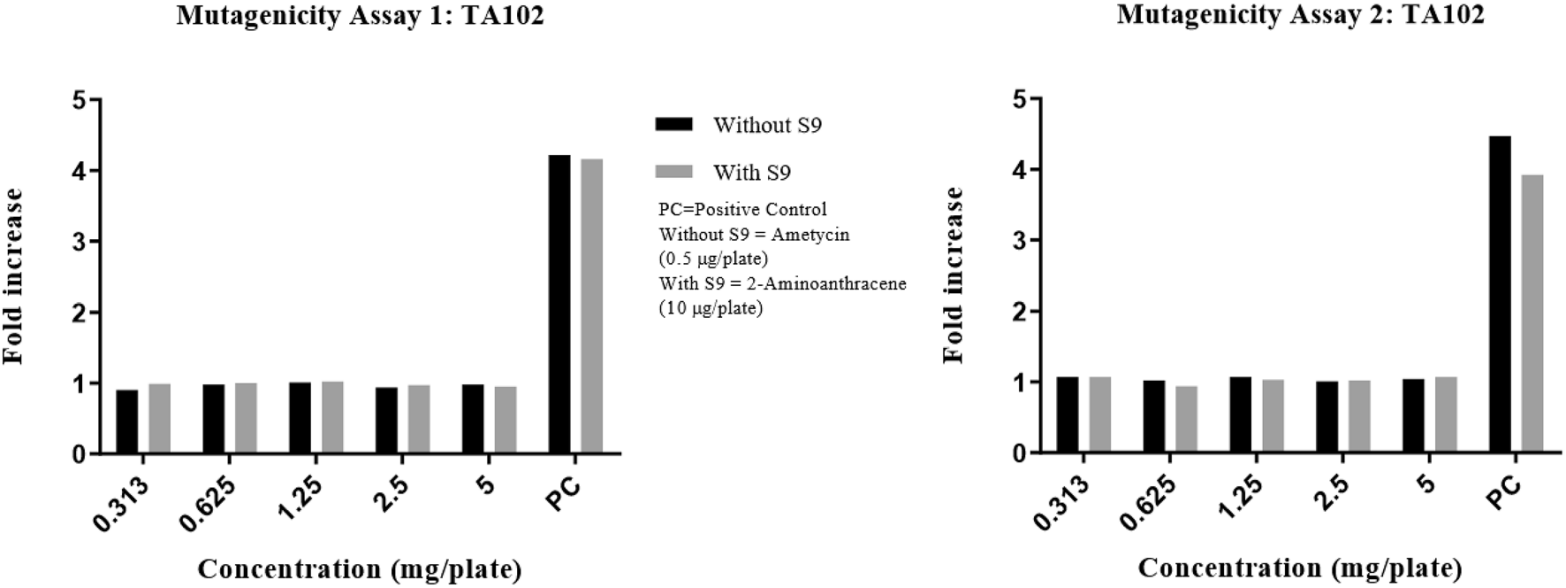
TA102: fold increase in revertant colony count (vs vehicle control): mutagenicity assay 1 and 2.

Precipitation was graded based on the amount of visible precipitate on the plate. Cytotoxicity was assessed in terms of diminution of background bacterial lawn, presence of micro colonies and/or reduction in the number of revertant colonies. In the DRF study, there were no signs of precipitation and cytotoxicity observed at the highest concentration (5 mg/plate) of test item (Synacinn) in any of the test conditions against TA100 strain. The fold increase in revertant colony count for each test items were illustrated in Fig. 1. The mutagenicity potential was evaluated on TA1537, TA1535, TA98, TA100, and TA102 strains. There was no significant increase in the revertant colony counts in test item treated plates for all strains in any of the conditions tested. The fold increase (vs vehicle control) seen in the revertant colony counts in the absence and presence of an exogenous metabolic activation system (S9) in the test item treated groups was 0.58–1.17 for TA1537, 0.64–1.28 for TA1535, 0.71–1.24 for TA98, 0.87–1.05 for TA100 and 0.90–1.07 for TA102 using the plate incorporation or pre-incubation methods (Fig. 2, 3, 4, 5, 6). The negative control counts fell within three times the standard deviation of the historical range for all the tester strains in the DRF and mutagenicity assays. The positive controls showed significant increases in revertant numbers in all the tester strains, confirming discrimination between different strains, and active S9 preparation. A summary of revertant colony counts obtained in DRF and mutagenicity assay 1 and 2 are presented in Tables 1 and 2.

**Table 1:** Summary of mean revertants—mutagenicity assay 1 by plate incorporation method.

Test item	Treatment (mg/plate)	TA1537	TA1535	TA98	TA100	TA102
		Number of revertants (mean ± SD)				
Organism/untreated control	0	13 ± 4.2	18 ± 5.0	54 ± 8.6	190 ± 10.2	473 ± 8.5
**Without S9**						
Vehicle control	0	12 ± 1.7	18 ± 3.2	58 ± 9.5	198 ± 25.1	538 ± 22.1
Synacinn	0.313	9 ± 1.7	19 ± 5.9	69 ± 11.5	207 ± 22.1	482 ± 11.4
	0.625	7 ± 2.6	21 ± 8.1	72 ± 10.0	177 ± 20.6	527 ± 29.3
	1.25	11 ± 4.4	22 ± 9.3	65 ± 15.5	190 ± 30.4	541 ± 10.6
	2.5	10 ± 2.5	18 ± 4.0	61 ± 18.0	191 ± 10.4	508 ± 31.5
	5	14 ± 5.1	23 ± 4.2	61 ± 13.1	186 ± 3.2	527 ± 24.0
S9 positive control (2-aminoanthracene)	5 (µg/plate)	–	–	–	192 ± 16.8	–
Positive control		ICR-191 (1 µg/plate)	Sodium azide (5 µg/plate)	2-Nitrofluorene (5 µg/plate)	Sodium azide (5 µg/plate)	Ametycin (0.5 µg/plate)
		173 ± 8.5	1035 ± 80.4	886 ± 32.2	1666 ± 95.0	1995 ± 107.8
**With S9**						
Vehicle control	0	14 ± 2.6	14 ± 4.2	50 ± 1.5	224 ± 11.0	518 ± 21.0
Synacinn	0.313	13 ± 2.9	12 ± 5.9	54 ± 4.4	215 ± 4.6	514 ± 15.6
	0.625	13 ± 2.3	11 ± 0.6	53 ± 10.3	197 ± 10.5	517 ± 67.1
	1.25	12 ± 0.6	11 ± 4.2	56 ± 9.6	210 ± 10.1	526 ± 67.4
	2.5	11 ± 4.2	9 ± 3.5	52 ± 9.6	221 ± 10.7	504 ± 17.2
	5	10 ± 0.6	9 ± 1.2	58 ± 5.0	215 ± 7.2	494 ± 38.6
S9 positive control (2-aminoanthracene)	5 (µg/plate)	225 ± 23.4	208 ± 45.6	1561 ± 94.5	2319 ± 292.4	–
	10 (µg/plate)	–	–	–	–	1966 ± 66.3

N = 3. None of the treatment plates showed any test item precipitation or cytotoxicity.

**Table 2:** Summary of mean revertants—mutagenicity assay 2 by pre-incubation method.

Test item	Treatment (mg/plate)	TA1537	TA1535	TA98	TA100	TA102
		Number of revertants (mean ± SD)				
Organism/untreated control	0	6 ± 2.9	17 ± 2.3	60 ± 6.5	135 ± 3.1	426 ± 77.7
**Without S9**						
Vehicle control	0	6 ± 2.1	17 ± 5.8	63 ± 6.7	170 ± 11.6	441 ± 10.7
Synacinn	0.313	6 ± 3.5	18 ± 2.1	57 ± 9.1	166 ± 34.7	473 ± 19.7
	0.625	5 ± 1.0	12 ± 2.3	46 ± 6.6	148 ± 10.1	451 ± 16.3
	1.25	6 ± 3.2	13 ± 3.2	53 ± 11.0	157 ± 30.0	472 ± 19.0
	2.5	6 ± 3.1	17 ± 8.7	45 ± 8.7	162 ± 11.7	444 ± 30.8
	5	7 ± 2.0	15 ± 2.1	75 ± 14.5	153 ± 26.0	458 ± 13.5
S9 positive CONTROL (2-aminoanthracene)	5 (µg/plate)	–	–	–	170 ± 11.9	–
Positive control		ICR-191 (1 µg/plate)	Sodium azide (5 µg/plate)	2-Nitrofluorene (5 µg/plate)	Sodium azide (5 µg/plate)	Ametycin (0.5 µg/plate)
		211 ± 14.0	1210 ± 13.5	943 ± 90.0	1541 ± 70.9	1943 ± 143.5
**With S9**						
Vehicle control	0	10 ± 3.8	8 ± 1.2	40 ± 6.1	180 ± 11.6	386 ± 36.3
Synacinn	0.313	10 ± 3.2	8 ± 2.9	36 ± 8.5	183 ± 18.3	413 ± 28.1
	0.625	8 ± 3.0	8 ± 2.3	34 ± 1.0	166 ± 37.2	362 ± 43.6
	1.25	9 ± 4.6	8 ± 3.2	37 ± 4.4	161 ± 1.0	399 ± 9.5
	2.5	8 ± 2.1	9 ± 3.6	35 ± 4.5	167 ± 7.8	392 ± 12.3
	5	7 ± 3.5	10 ± 4.7	41 ± 7.8	167 ± 12.5	414 ± 15.5
S9 positive control (2-aminoanthracene)	5 (µg/plate)	192 ± 19.9	181 ± 28.1	1459 ± 160.0	1755 ± 87.3	–
	10 (µg/plate)	–	–	–	–	1670 ± 163.7

N = 3. None of the treatment plates showed any test item precipitation or cytotoxicity.

### Bone marrow micronucleus test

Doses tested in the preliminary study were well tolerated with no evident signs of toxicity. There were no treatment-related clinical signs, body weight reduction, or bone marrow toxicity observed in any of the treatment group animals. Summary of the body weights throughout the treatment (Day 1–3) and the percentage of the body weight gain was illustrated in Supplementary Fig. S1. Therefore, the highest recommended dose of 2000 mg/kg/day was selected as the maximum dose for the definitive study. Two lower dose levels of 1000 and 500 mg/kg/day were selected for determining the dose–response. In the definitive study, there was no reduction in the PCE/E ratio observed across the treatment groups and was comparable with the vehicle control group. Statistically significant reduction in PCE/E ratio (18.33%) was observed in the positive control group treated with cyclophosphamide monohydrate (25 mg/kg) when compared with the vehicle control group. There was no statistically or biologically significant increase in the MN PCE counts across the test item treatment groups. The mean MN PCE count for the vehicle control was 4.2 while for the test item treated groups at 500, 1000, and 2000 mg/kg/day, the mean MN PCE count was 3.5, 3.8, and 3.8, respectively. The positive control (cyclophosphamide monohydrate) group showed a statistically and biologically significant increase in the incidence of MN PCE compared to the vehicle control group. The mean MN PCE count for the animals treated with cyclophosphamide monohydrate was 89.8 (Table 3). The mean frequency of MN PCE per 1000 PCEs obtained in the vehicle control group (1.01) of this study was within three times the standard deviation of the historical vehicle control data of the test facility.

**Table 3:** Summary of mean PCE/E, PCE/NCE and MN PCE.

Group no	Parameters Preliminary study		Group no	Parameters Definitive study		
	PCE/E	PCE/NCE		PCE/E	PCE/NCE	MN PCE
G1	0.63 ± 0.095	1.80 ± 0.654	G6	0.60 ± 0.025	1.52 ± 0.156	4.2 ± 1.33
G2	0.64 ± 0.006	1.77 ± 0.035	G7	0.63 ± 0.035	1.72 ± 0.230	3.5 ± 0.84
G3	0.65 ± 0.045	1.86 ± 0.366	G8	0.59 ± 0.040	1.43 ± 0.225	3.8 ± 1.17
G4	0.65 ± 0.029	1.92 ± 0.224	G9	0.60 ± 0.027	1.51 ± 0.157	3.8 ± 1.47
G5	0.64 ± 0.026	1.79 ± 0.207	G10	0.49 ± 0.026	0.94 ± 0.094	89.8 ± 19.16

*PCE* polychromatic erythrocytes, *NCE* normochromatic erythrocytes, *E* total erythrocytes, *G1* vehicle control: 0 mg/kg/day, *G2* Synacinn: 300 mg/kg/day, *G3* Synacinn: 600 mg/kg/day, *G4* Synacinn: 1000 mg/kg/day, *G5* Synacinn: 2000 mg/kg/day, *G6* vehicle control: 0 mg/kg/day, *G7* Synacinn: 500 mg/kg/day, *G8* Synacinn: 1000 mg/kg/day, *G9* Synacinn: 2000 mg/kg/day, *G10* cyclophosphamide monohydrate: 25 mg/kg.

### Safety pharmacology

**Figure 7:**
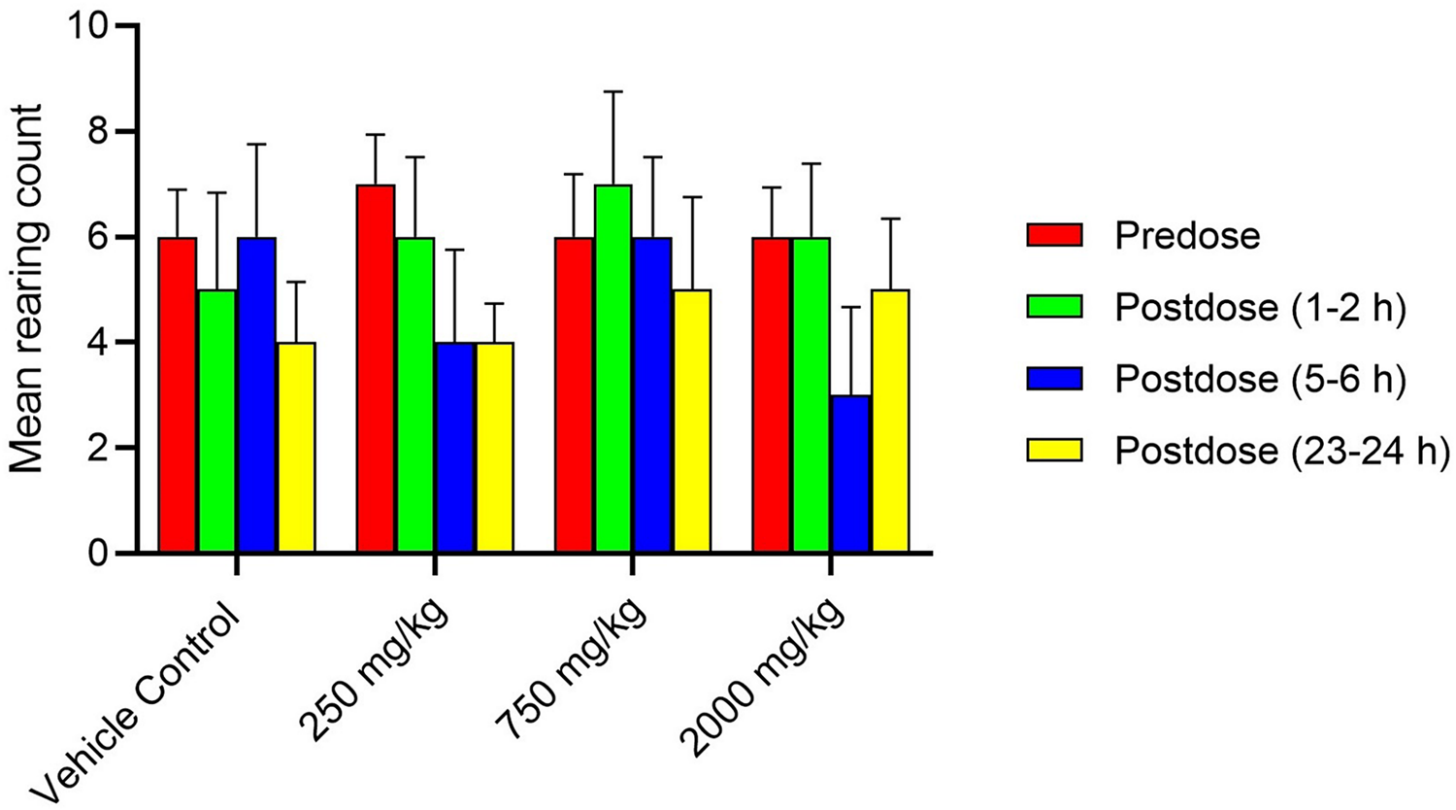
Rearing count of Sprague Dawley rats at predose and postdose. Data represent the mean of respective group measurements and are expressed as mean ± SEM.

**Figure 8:**
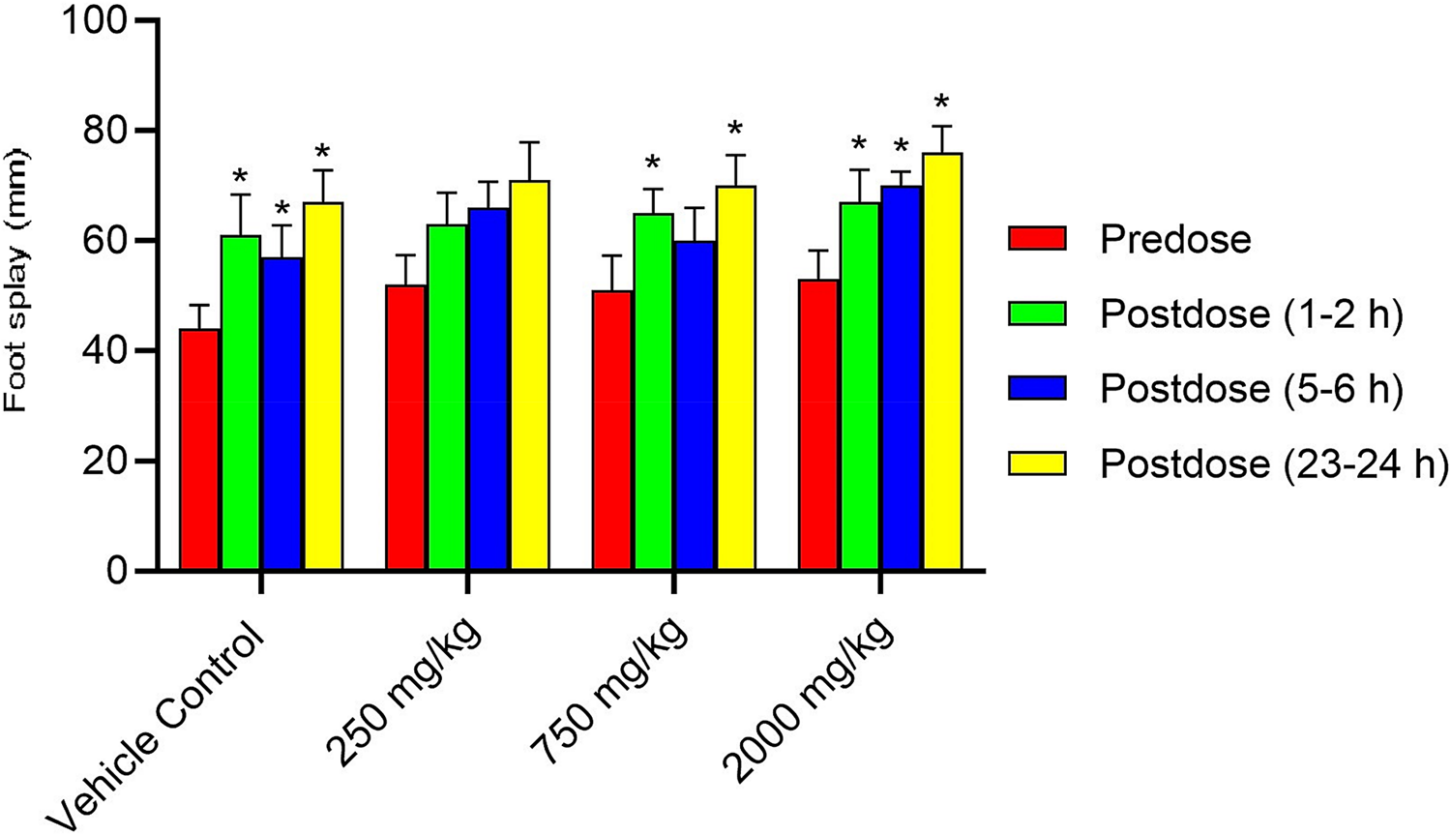
Landing foot splay of Sprague Dawley rats at predose and postdose. Data represent the mean of respective group measurements and are expressed as mean ± SEM.

**Figure 9:**
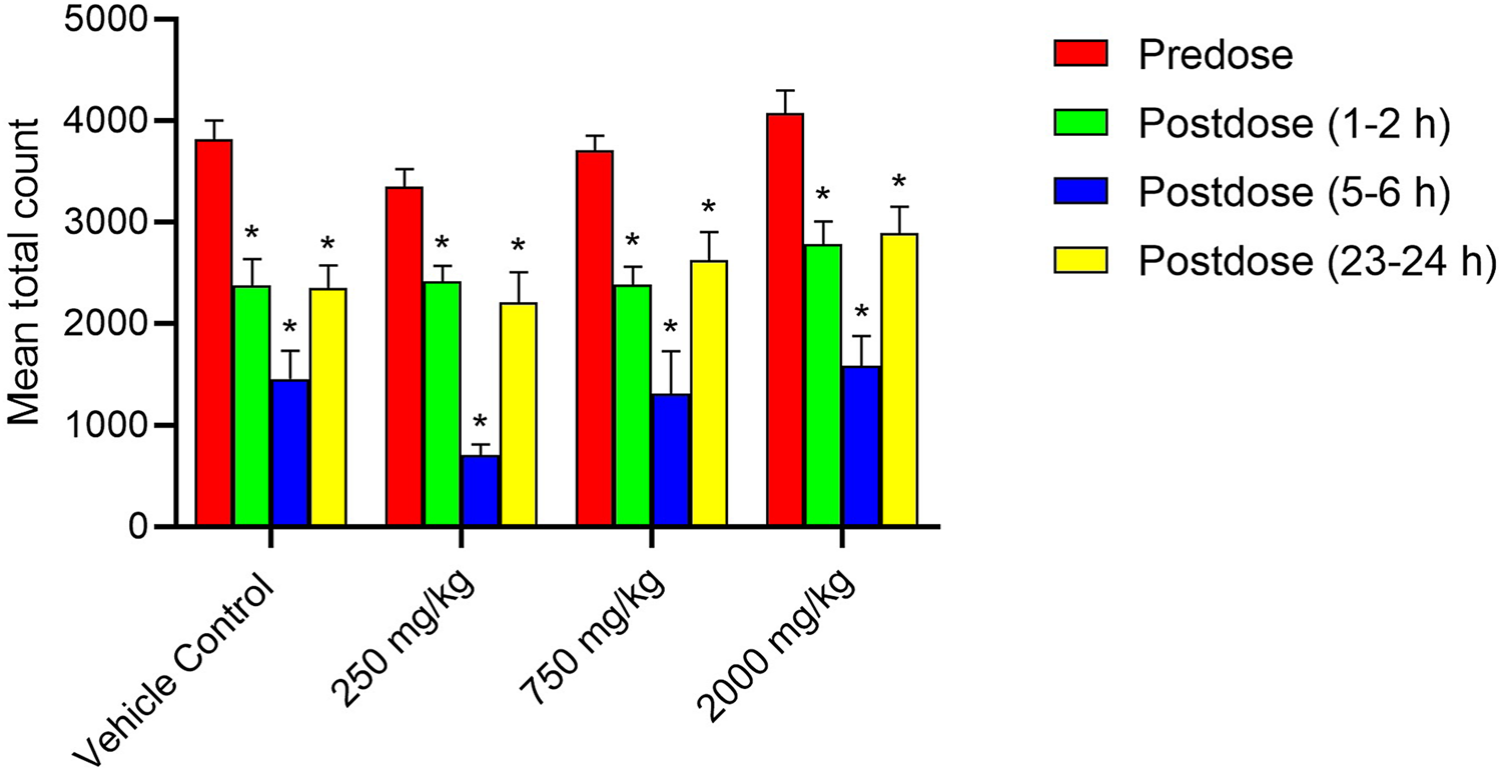
Total count of motor activity in Sprague Dawley rats at predose and postdose. Data represent the mean of respective group measurements and are expressed as mean ± SEM.

**Figure 10:**
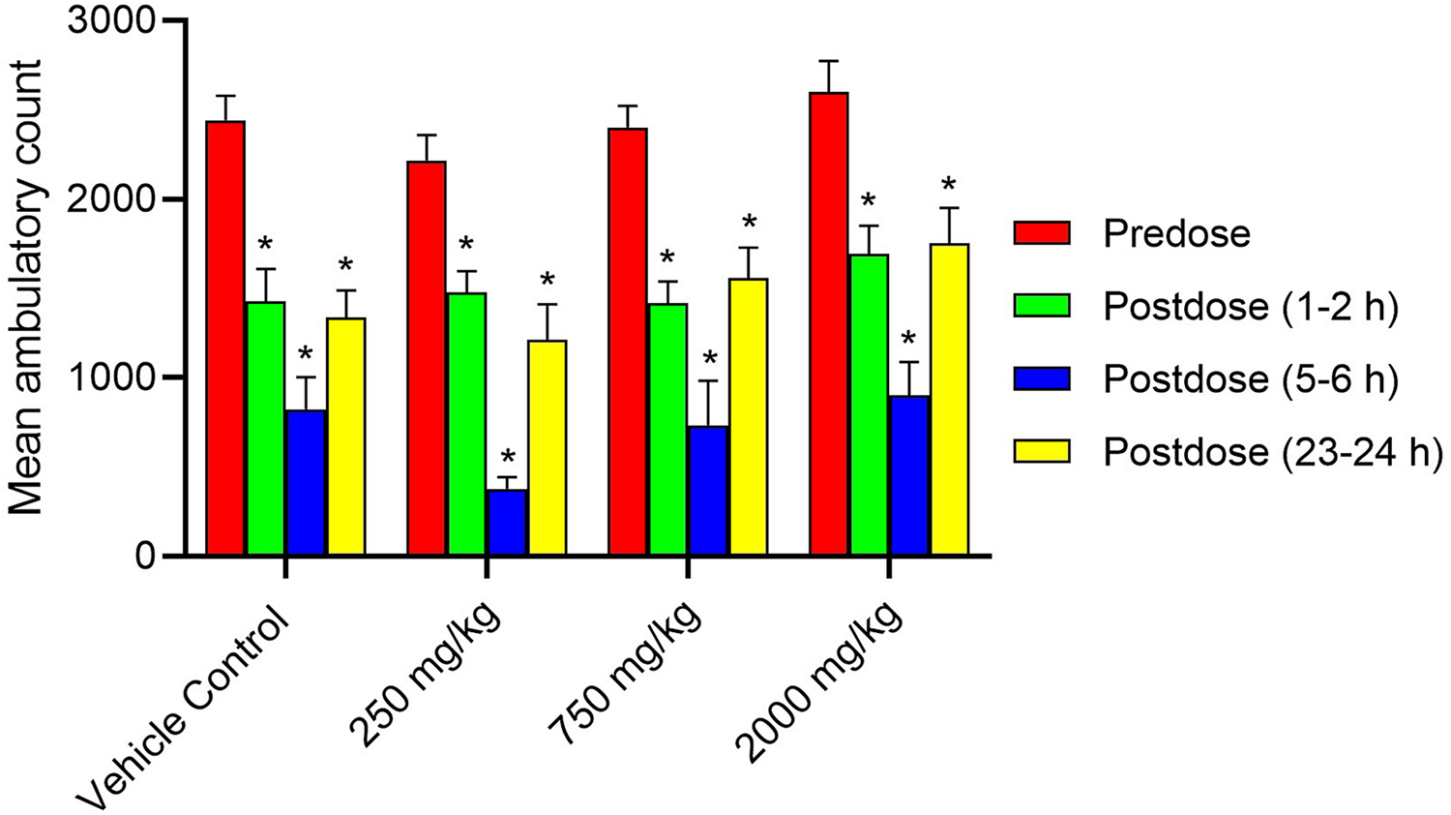
Ambulatory count of motor activity in Sprague Dawley rats at predose and postdose. Data represent the mean of respective group measurements and are expressed as mean ± SEM.

**Figure 11:**
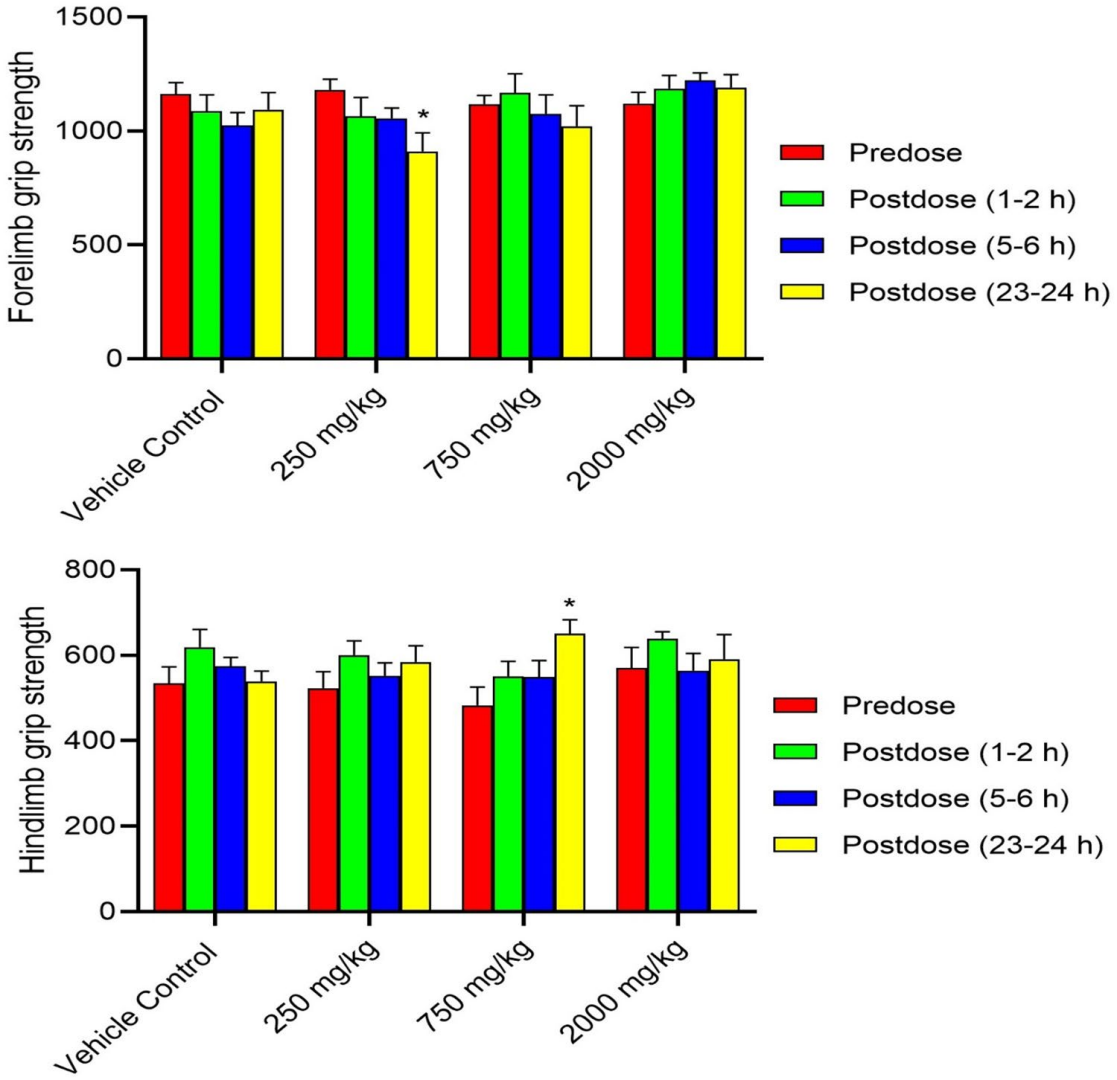
Forelimb and hindlimb grip strength of Sprague Dawley rats at predose and postdose. Data represent the mean of respective group measurements and are expressed as mean ± SEM.

Superimposed records of hERG potassium currents obtained for the 1 mg/mL of Synacinn, vehicle control (External solution), and positive control (1 µM Verapamil) are shown in Supplementary Fig. S2. Table 4 summarizes the inhibition effect of the test items on hERG potassium current. Synacinn did not produce any significant inhibition of hERG potassium current up to the highest tested concentration of 1 mg/mL. The positive (verapamil) and negative control (aspirin) produced 79% and 6% inhibition of hERG current, respectively, thus confirming the sensitivity of the assay to the hERG current inhibition. Supplementary Table 1S summarizes the Functional Observational Battery (FOB) findings for all time points and animal groups. There was no Synacinn related effect on the rearing counts (Fig. 7), landing foot splay (Fig. 8), total counts (Fig. 9) and ambulatory counts (Fig. 10) in motor activity, changes in forelimb and hindlimb (Fig. 11) grip strength, body temperature (Supplementary Figure S3) and weight (Supplementary Figure S4), at measurements up to 2000 mg/kg in rats.

**Table 4:** Effect of Synacinn, positive and negative control on hERG potassium current.

Test item/control	Concentration	% inhibition	N	Precipitation score*
Synacinn	1 mg/mL	2.7 ± 2.5 (SEM = 1.5)	3	0
Verapamil (positive)	1 µM	79	1	0
Aspirin (negative)	10 1 µM	6	1	0

N = Number of cells tested; *SEM* standard error of mean; *A precipitation score of 2 and above is considered unsuitable for testing.

## Discussion

In the early 1970s, Ames assay was introduced by Ames and colleagues and is presently known as the standard mutagenic test for determining the genotoxicity of drugs, particularly for the preliminary screening test^14^. Multiple strains of *S. typhimurium* used in Ames assay were genetically defective whereby the strains were not able to produce the essential amino acid, histidine, and can only grow in the histidine-rich medium. However, the introduction of mutagens or carcinogens to the strains may reverse their defective function to normal and thus allowing them (revertant colonies) to grow in the standard medium. This further explains the term used for this test which is also known as the bacteria reverse mutation test. An exogenous metabolic enzyme was also incorporated into this test since bacteria do not produce enzymes required for the metabolic activation of the promutagens. The addition of this enzyme (S9) may determine the requirement for the metabolic activation of the test item in its mutagenic expression^15^. Based on the present study, Synacinn at all concentration tested did not significantly increase the number of revertant colonies of *S. typhimurium* for all strains either in the presence or absence of S9, which indicate that Synacinn is a non-mutagenic agent.

A mammalian in vivo micronucleus test is used for the detection of damage induced to the chromosomes or the mitotic apparatus of erythroblasts by the test item. This test evaluates micronucleus formation in erythrocytes sampled either in the bone marrow or peripheral blood cells of animals. The micronucleus test identifies substances that cause cytogenetic damage which results in the formation of micronuclei containing either lagging chromosome fragments or whole chromosomes. When a bone marrow erythroblast develops into an immature erythrocyte (polychromatic erythrocyte—PCE), the main nucleus is expelled and the micronucleus remains behind in the cytoplasm^16^. An increase in the frequency of micronucleated immature erythrocytes (MN PCE) in treated animals is an indication of induced structural or numerical chromosomal aberrations. In addition, a decrease in PCE/NCE ratio is an indicator of toxicity involving bone marrow cell formation^16,17^. Based on the in vivo micronucleus test of Synacinn, there was no significant increase in the MN PCE frequency as well as no decrease in the PCEs/NCEs ratio. This finding may suggest that Synacinn may not pose any toxicity or clastogenicity potential.

Studies on the mutagenicity potential of all the individual herbs that are formulated in Synacinn had been previously reported. Extracts of *A. paniculata* and *O. stamineus* were reported to demonstrate no mutagenicity or clastogenicity potential in the Ames assay as well as bone marrow micronucleus test^18,19^. Mutagenicity study of *C. zeylanicum* had also been done previously using the Ames and rec assay whereby the results showed no mutagenic effect either in the presence or absence of S9 in both assays^20,21^. As for *S. polyanthum*, the genotoxicity study of its extract was evaluated using a comet assay and the result showed no genotoxic effect that can cause DNA damage^22^. Meanwhile, an extract of *C. xanthorrhiza* was reported to show anti-mutagenic activity through the Ames assay^23^. Thus, it may suggest that all the five herbs formulated in Synacinn are safe and demonstrate no genotoxic potential either individually or in the form of mixtures.

Cardiac electrical activity is regulated by ion channels whereby one of them is the hERG potassium channel, the main control of action potential (AP) duration^24^. The evaluation of the hERG channel blockage potential is essential for the drug development process as the channel blockage caused prolongation of QT interval (duration from ventricular depolarization to repolarization) which may lead to cardiac arrhythmias or irregular heartbeat^25^. In a previous study, inhibition on the hERG potassium channel of a single plant extract using *Xenopus* oocytes had been reported on *Cinnamomum zeylanicum* Nees Lauraceae’s bark, with 64.5% inhibition observed in a voltage-clamp assay. However, by removing tannins from the extract, the inhibition on the hERG channel was found to be significantly lower. Thus, the study suggested that tannin was responsible for the observed inhibitory activity. Nonetheless, it can be considered as negligible as tannin has low bioavailability in the in vivo model^26,27^. Based on the present study, human embryonic kidney (HEK) 293 cells were used to evaluate the inhibition activity of a polyherbal formulation, Synacinn, on the hERG potassium channel. IC_50_ calculation was not done as Synacinn did not produce any significant inhibition of hERG current at the highest tested concentration of 1 mg/mL. It was found that Synacinn did not block the hERG potassium channel which suggests that Synacinn does not possess any adverse cardiotoxicity effect.

The first-tier safety of FOB test was done to determine the neurotoxicity potential of Synacinn. The multi-parameter of neurobehavioural evaluation in the FOB test consists of autonomic, neuromuscular, sensorimotor, and behavioural observations. The neurobehavioural assessment is crucial at an early stage of drug discovery as some neurotoxic agents may affect behaviours with no central nervous system (CNS) lesion manifested^28^. Based on the observation, no mortality/moribundity and neurobehavioral changes were observed on the Sprague Dawley rats up to 2000 mg/kg. Statistically significant (*p* value ≤ 0.05) increase in hindlimb foot splay, at 750 mg/kg (post dose 1–2 h and 23–24 h) and at 2000 mg/kg (post dose 1–2 h, 5–6 h and 23–24 h) dose groups were considered as incidental findings as similar increase were also observed in vehicle control groups at all the post dose evaluations as compared to predose values. Meanwhile, statistically significant (*p* value ≤ 0.05) decrease in the forelimb grip strength at 250 mg/kg and increases in the hindlimb grip strength at 750 mg/kg for 23–24 h post dose as compared to the predose assessment were also considered as incidental findings in the absence of such changes in the higher dose group animals. The total counts and ambulatory motor activity counts of all the treated groups rats were significantly decrease (*p* value ≤ 0.05) during post dose observation time points as compared to predose counts. These changes were not treatment related and considered to be biologically insignificant as similar reductions in post dose were also noted in the vehicle control group animals in comparison to the predose values.

The biological activities of herbal medicines are often due to interactions between active compounds in the herbal formulation. These interactions could be synergistic, additive and antagonistic. Synergistic interaction may increase efficacy as well as minimize toxicity of the herbal medicine, while additive interactions may create a summation effects^29^. In the present study, Synacinn which was a mixture of five plants showed no toxicity in the in vitro and in vivo tests. This could be due to synergistic interaction between the five biomarkers (rosmarinic acid, gallic acid, curcumin, catechin, and andrographolide) where some of these biomarkers could have counteracted the toxicity while maintaining the therapeutic effect of the herbal formulation. Previous studies could support this hypothesis as rosmarinic acid was found to increase the efflux activity of transporters such as P-gp and MRP2^30^. This efflux activity is known to enhance the detoxification by preventing the intracellular accumulation of toxic chemicals^31^. Meanwhile, the therapeutic effect could have been maintained by curcumin which was previously reported as an effective inhibitor for the efflux transporters and able to increase the blood concentration of the therapeutic agents^31^.

## Conclusions

Synacinn is non-mutagenic at concentrations up to 5 mg/plate in the presence and absence of an exogenous metabolic activation system (S9) against the *Salmonella typhimurium* strains of TA1537, TA1535, TA98, TA100 and TA102. It did not induce clastogenicity at the maximum tolerated dose level of 2000 mg/kg/day in Sprague Dawley rat bone marrow. Meanwhile, in the pharmacological safety tests, Synacinn was observed to have no significant inhibitory effect on the hERG potassium channel and absence of any abnormal neurobehavioral changes in the Sprague Dawley rats up to the highest tested dose of 2000 mg/kg. Thus, Synacinn may not have any potential mutagenicity, cardiotoxicity, and neurotoxicity effects.

## Materials and methods

### Materials

Synacinn powder was supplied by Proliv Life Sciences Sdn Bhd. Adventol (50% in purified water) and purified water were used as vehicles. The positive controls used in the Ames assay were 2-Nitrofluorene, Sodium azide, ICR-191, Ametycin, and 2-Aminoanthracene. As for the MN test, cyclophosphamide monohydrate (Sigma-Aldrich) was used as the positive control. Verapamil (1 µM, Fluka), Aspirin (10 µM, Sigma Life Sciences) and 10 mM HEPES buffer were control items used for safety pharmacology tests.

### Strain maintenance and revival

*Salmonella typhimurium* strains of TA102, TA100, TA98, TA1535, and TA1537 were used in this experiment as recommended by the OECD 471 and ICH S2 (R1) guidelines^32,33^. All the strains were originally obtained from Molecular Toxicology, INC. Boone, NC, USA. The genotyping was verified by determining its sensitivity to UV radiation, histidine requirement, rfa mutation, and resistance to ampicillin/ tetracycline. Only qualified batches of strains were employed in the experiments. All strains were maintained in Cryovials as frozen stocks and stored in a − 70 °C freezer. The frozen cultures were thawed prior to use and added into Oxoid Nutrient Broth (ONB-2) containing flask. The inoculated flask was incubated overnight (15–16 h) in a shaking incubator at 37 °C and 110 rpm. The incubated cultures with an optical density of 10^9^ CFU/mL were used and measured by spectrophotometer at 650 nm. The viable count of colonies on nutrient agar plates was done to determine the actual cell titers.

### Metabolic activation system

#### Mammalian liver post mitochondrial fraction (S9)

In the metabolic activation system, mammalian liver post mitochondrial fraction (MOLTOXTM S9) prepared from Sprague Dawley male rats (Aroclor 1254-induced) was obtained from Molecular Toxicology Incorporated, USA. All batches of MOLTOXTM S9 were kept frozen at − 70 ± 10 °C and thawed prior to use. Every batch was inspected by the manufacturer for protein content, ability to convert known promutagens to bacterial mutagens, sterility and cytochrome P450 catalysed enzyme activities^14,34,35^.

#### S9 mix preparation

A freshly prepared S9 mix was done for each assay by mixing 10% v/v of S9 fraction with the required cofactors (1:9 mixture ratio) which were stored at − 20 °C prior to use. The prepared S9 mix was placed in the ice throughout the experiment and the remains were discarded. The composition of the cofactors used was as described in the previously reported data^36^.

### Plating procedure

#### Plate incorporation method

Plate incorporation method was done by sequentially adding Synacinn or positive or vehicle control solution (0.05/0.1 mL), phosphate buffer solution (0.5 mL), and bacterial culture (0.1 mL) into 2 mL of molten agar (45 ± 2 °C; supplemented with 10% v/v, 0.5 mM Histidine-Biotin solution), followed by pouring the mixtures into pre-labelled minimal glucose agar plates for the treatment without metabolic activation. Once the agar completely solidified, the plates were inverted and incubated. As for the metabolic activation treatment, the S9 cofactor mix (0.5 mL) was added instead of phosphate buffer. For untreated (i.e. organism) control, respective strain (0.1 mL) was added to the molten agar (2 mL) and poured into pre-labelled minimal glucose agar plates. The plate incubation was done for 48 to 72 h at 37 ± 1 °C.

#### Pre-incubation method

In the pre-incubation method, all the steps for sequential mixture addition were as described in the routine plate incorporation procedure, followed by a pre-incubation step in a shaking water bath (75 rpm) for 20 min at 37 ± 1 °C prior to the addition of 2 mL of molten agar (45 ± 2 °C) into the mixture. The mixture was then poured into pre-labelled minimal glucose agar plates and further incubated for 48–72 h at 37 ± 1 °C.

### Study design of Ames assay

A dose range finding (DRF) study was designed to evaluate the cytotoxicity and precipitation of test items, with an aim to select doses for the mutagenicity assay. The DRF study was done using the plate incorporation method on one of the *S. typhimurium* strains, TA100, at eight doses (5, 2.5, 1.25, 0.625, 0.313, 0.156, 0.078, and 0.039 mg/plate) of test items, with and without metabolic activation (10% S9 cofactor mix) in triplicate. Based on the DRF data, mutagenicity assays 1 and 2 were conducted using *S. typhimurium* strains of TA98, TA102, TA100, TA1535, and TA1537 at five selected doses (5, 2.5, 1.25, 0.625, and 0.313 mg/plate), with and without metabolic activation system (10% S9 cofactor mix). Mutagenicity assay 1 was done using the plate incorporation method while mutagenicity assay 2 was done using the pre-incubation method. In the DRF and mutagenicity assays, each treatment plate was assessed for precipitation and cytotoxicity after incubation for 48–72 h. Revertant colonies were counted manually. The fold increase in revertant colony counts for each test item treatment versus the vehicle control group was determined to assess the mutagenic potential of the test item. The assays were carried out following the OECD 471, ICH S2 (R1), and EMEA guidelines^32,33,37^.

### Study design of bone marrow micronucleus test

The in vivo test was conducted and designed based on the ICH S2 (R1) and OECD guidelines^33,38,39^. A stratified randomized design (Microsoft Excel^®^) was used in the grouping and allocation of healthy rats (age of 8–10 weeks) to the respective treatment. Rats were fed with the gamma-irradiated commercial pellet diet, ad libitum, throughout the experimental period. The mean of body weights for each group of rats was confirmed prior to the treatment so that there were no significant differences (p ≤ 0.05) among each other with acceptance criterion for the variation in the mean body weight for each sex must be less than 20%. All experiments were conducted with the approved procedures of the Institutional Animal Ethics Committee (IAEC).

#### Preliminary study of micronucleus test

A preliminary study was conducted at doses of 0 (vehicle control: Group 1), 300 (Group 2), 600 (Group 3), 1000 (Group 4), and 2000 (Group 5) mg/kg/day of Synacinn in 3 males and 3 females. This study aimed to evaluate the bone marrow cytotoxicity as well as general toxicity of Synacinn. This study also provided a basis in the dose selection for the definitive study of bone marrow MN test. An oral gavage administration of Synacinn to Sprague Dawley rats was conducted for two consecutive days at 24 h intervals. Based on the body weight of rats on day one of study, the dose volumes were calculated. The dose volumes for 300, 600, and 1000 mg/kg/day treatment were 10 mL/kg body weight while for the vehicle control and 2000 mg/kg/day treatment, the dose volume was 20 mL/kg body weight. Several observations such as body weight (pre dose on day 1 and prior to sacrifice), mortality/moribundity (twice daily), and clinical signs (pre dose and post dose) were recorded. The rats were sacrificed approximately 24 h from the last treatment. The bone marrow smears were prepared from rat femurs. The ratio of Polychromatic Erythrocytes: total erythrocytes (PCE/E) was determined by microscopic analysis of the bone marrow smears.

#### Definitive study of micronucleus test

In the definitive study of bone MN test, the clastogenicity potential of Synacinn was evaluated. Based on the preliminary study, 500 (Group 7), 1000 (Group 8), and 2000 (Group 9) mg/kg/day were selected as treatment doses in the definitive study and the animal group without treatment was assigned as vehicle control (Group 6). As no gender-specific difference was observed in terms of toxicity in the preliminary study, the definitive study was conducted using male animals only (6 rats/group). The oral gavage administration of Synacinn to Sprague Dawley rats was conducted under fed conditions for two consecutive days at 24 h intervals. Meanwhile, for the positive control, a single intraperitoneal injection was done only on Day 2 of treatment at 25 mg/kg (Group 10). The observation on animal body weight, mortality/moribundity, and clinical signs were recorded as in the preliminary study. The PCE/E ratio was determined to evaluate the bone marrow toxicity. The Micronucleated Polychromatic Erythrocytes (MN PCE) counts were also recorded.

### Bone marrow evaluation

#### Animal sacrifice and bone marrow collection

CO2 asphyxiation of rats was done to sacrifice the animals at approximately 18–24 h after the second dosing. Bone marrow collection from animals was conducted by femurs isolation and cutting open the epiphyses, followed by flushing out the bone marrow with fetal bovine serum and collected into centrifuge tubes. The bone marrow cells were centrifuged at 1000 rpm (5 min) at room temperature and pellets were harvested. A homogenous bone marrow suspension was prepared and spread (5–10 µL) onto a clean glass slide which was further fixed by methanol. There were two slides of smears prepared for the preliminary study and four slides of smears for the definitive study of which each slide was coded prior to analysis.

#### Bone marrow toxicity by PCE/E ratio determination

The proportion of immature erythrocytes to total erythrocytes (immature + mature) was determined to evaluate the bone marrow toxicity. May Grunwald’s Giemsa was used to stain the methanol fixed slides. Each animal in the preliminary and definitive studies were evaluated for at least 500 erythrocytes. A decrease in the PCE/E ratio as compared to the respective vehicle control was regarded as a bone marrow toxicity measurement.

#### Micronucleated PCEs (MN PCEs) determination

Acridine Orange was used in the staining of methanol fixed slides for determining the MN PCEs count. The selected area of cells was viewed using a fluorescent microscope with medium magnification. The PCEs (≥ 4000) for each animal was scored for the estimation of micronucleated cells. Several observations were recorded for each animal such as the number of PCE differentiated and PCE with micronuclei, total RBC/erythrocytes, mean and SD of PCE with micronuclei, and PCE: Total RBC ratio.

### hERG assay

#### Test formulation preparation

Synacinn was dissolved in 10 mM HEPES buffer to give a concentration of 1 mg/mL. An initial concentration of 1 mg/mL (highest concentration) was tested and based on the response obtained, further test concentration was decided. The prepared solution was vortexed and filtered prior to testing.

#### Cell culture

HEK 293 cells (Merck Millipore, MA, USA) stably expressing hERG potassium channel were cultured in Dulbecco’s Modified Eagle Medium: Nutrient Mixture F-12 (DMEM/F-12) in standard tissue culture flasks and passage every 2–3 days at a confluency of 60–80%. Cells were harvested using Trypsin as a detaching agent for electrophysiology procedures.

#### Patch clamp solution

Internal solution: 20 mM EGTA free acid, 8 mM NaCl, 50 mM KCl, 60 mM KF, 10 mM HEPES free acid, Osmolarity 280–290 mOsm/kg, pH(KOH) 7.2. External solution: 4 mM KCl, 2 mM CaCl_2_·2H_2_O, 1 mM MgCl_2_·6h_2_O, 5 mM Dextrose monohydrate, 140 mM NaCl, 10 mM HEPES free acid, Osmolarity 295–305 mOsm/kg, pH(NaOH) 7.4. Seal enhancer solution: 3 mM KCl, 35 mM CaCl_2_·2H_2_O, 10 mM MgCl_2_·6h_2_O, 80 mM NaCl, 10 mM HEPES sodium salt, Osmolarity 295–305 mOsm/kg, pH(HCl) 7.4.

#### Electrophysiology procedures

A semi-automatic of Port-A-Patch (Nanion Technologies GmBH, Germany) system was used in the electrophysiology procedures. The current recording was conducted at room temperature. Cells were exposed to each test concentration for three minutes or till a steady-state block was reached. The voltage protocol was as follows: − 40 mV subtraction pulse for 0.5 s; + 40 mV activation prepulse for 2 s and − 80 mV holding potential repeated at every 10 s. The steady-state current after the application of the vehicle was considered as baseline (control current). Steady-state current obtained at the end of each test concentration addition was used to calculate the % hERG current inhibited at each concentration. The average current of the last 3–5 sweeps of acceptable quality was considered for calculation of % inhibition. Sweeps with artifacts and noise were omitted from the calculation.

### Functional observation battery (FOB)

#### Test formulation preparation

For assessing neurobehavioral effects in the Sprague Dawley rats, the dose levels of 0 (vehicle control), 250, 750, and 2000 mg/kg were selected. The doses were within the same dose range as employed in the previous acute oral toxicity study on Sprague Dawley rats. The highest dose was the limit dose and also the maximum tolerated dose (MTD) based on the results in the toxicity study on the Sprague Dawley rats^12^. The lower doses of 250 and 750 mg/kg were selected to assess the dose response. The appearance and pH of the formulations on the day of dosing were documented.

#### Animal care and maintenance

A total of 24 female Sprague Dawley rats were obtained from Hylasco Biotechnology Pvt Ltd, Hyderabad, India. They were weighed and acclimatized for 6 days. During the acclimatization period, animals were observed for mortality/moribundity (twice daily) and clinical signs (once daily). On the day of grouping, animals were again weighed and examined for general health. The rats’ age at the start of treatment was 8–9 weeks. Rats were housed in the Individually Ventilated Cages (IVC) and fed with the gamma-irradiated commercial pellet diet (Altromin, Germany), ad libitum, throughout the experimental period in an environmentally controlled air-conditioned room at 22 ± 3 °C, 30–70% of humidity.

#### Grouping and allocation of animals

Healthy rats were allocated to different treatment groups by a stratified randomized design using Microsoft Excel^®^. The mean body weights of each group before the start of the treatment were not statistically significant from each other (variation was less than 20% of the mean body weight of each sex). The animals were allocated to different groups as in Supplementary Table 2S.

#### Blinding of animals for FOB assessment

To avoid bias, until the trial outcome was known, information about the animal number and group number was masked from the trained FOB observer. This was done on the day of dose administration prior to predose observation by a person who was not involved in the FOB observation. Each animal was assigned a unique code. Animal cage cards were replaced with new cage cards containing blinded unique codes for the animal present in the cage. The order of testing was randomized among study groups and testing was performed in an area where sound disturbances were minimal. The coding procedure was captured in the raw data by the person not involved in FOB observation. At the end of observation, the identity of treatment to each animal was revealed for data compilation purposes.

#### Treatment

All rats were dosed on the same day. Rats were treated with a single dose of vehicle control or test item formulations orally by gavage on the scheduled day of dosing. Dose administration was carried out using a stainless-steel gavage needle fitted onto a disposable plastic syringe. The vehicle control and the test item formulations were continuously stirred using a magnetic stirrer throughout the dosing procedure. The vehicle control or the test item formulations were administered at a constant dose volume of 10 mL/kg and the individual dose volume was calculated based on the body weight of the animal measured on the day of dosing. Care was taken to avoid unintentional aspiration of the formulation into the airways.

#### Parameters observation

FOB tests were performed at predose and between 1–2, 5–6, and 23–24 h post-dosing. The time points mentioned were the starting time of observation and the observations were continued till the completion of the battery tests. The order of tests began with those requiring no or minimal handling to those requiring more manual manipulation. Animals were observed for a total of 44 parameters as tabulated in Supplementary Table 3S.

#### Euthanasia

At the end of the experiment, all the animals were euthanized by CO_2_ asphyxiation, and carcasses were disposed of following applicable Test Facility SOPs.

### Regulatory compliance

The study design and scope were based on the guideline of Assessment of Genotoxicity of Herbal Substances/Preparations (EMEA/HMPC/107079/2007), ICH S2 (R1) Current step 4 version: Guidance on Genotoxicity Testing and Data Interpretation for Pharmaceuticals Intended for Human Use, and ICH S7A: Safety Pharmacology Studies for Human Pharmaceuticals, Current Step 4 version. This study was conducted in compliance with Organization for Economic Co-operation and Development (OECD) Principles of Good Laboratory Practice. As for the animal ethics approval, this study was conducted by following the procedures approved by the Institutional Animal Ethics Committee of APSL, Hyderabad (IAEC Reference No.: ADTL/SE/005-07/11-2019).

### Statistical analysis

Statistical analysis was done using Sigma Plot^®^, Version 12.5, and a *p* value ≤ 0.05 was considered statistically significant. Data were categorized into Measurements, Count data, Rank order data, and Quantal data. For measurements and count data, intragroup comparisons were done using RM ANOVA followed by Bonferroni test or RM ANOVA on Ranks using predose as comparison factor. Quantal data analysis by ‘Fisher exact test’ (using a 2 × 2 contingency table) and Rank order data analysis was not performed as no differences in responses (present/absent) or ranks for relevant parameters were observed in postdose observations as compared to predose observations (intragroup comparisons) and in the test item treated animals as compared to vehicle control animals (intergroup comparisons).

### Ethics declarations

The authors declare that the article content was composed in the absence of any commercial or financial relationships that could be construed as a potential conflict of interest. As for the animal ethics approval, this study procedures was approved by the Institutional Animal Ethics Committee of APSL, Hyderabad (IAEC Reference No.: ADTL/SE/005-07/11-2019). All animal studies were conducted in accordance with the IAEC guidelines. This study was also conducted in compliance with the ARRIVE guidelines.

## Supplementary Information

Supplementary Information.

## Data Availability

The datasets used and/or analysed during the current study available from the corresponding author on reasonable request.
