# Supplementary material for: Mutagenicity and safety pharmacology of a standardized antidiabetic polyherbal formulation
Source: Sci Rep. 2022 May 3;12:7127. doi: 10.1038/s41598-022-11243-3 (PMC9065066; doi:10.1038/s41598-022-11243-3)
Supplement: Supplementary file 1 — Supplementary Information. [file 41598_2022_11243_MOESM1_ESM.docx]

Table 1S. Summary of Functional Observational Battery (FOB) findings

| **Group** | | **Vehicle control** | | | | **Synacinn™ (mg/kg)** | | | | | | | | | | | |
| --- | --- | --- | --- | --- | --- | --- | --- | --- | --- | --- | --- | --- | --- | --- | --- | --- | --- |
|  |  |  |  |  |  | **250** | | | | **750** | | | | **2000** | | | |
| **Time point** | | **Pre dose** | **1-2 h** | **5-6 h** | **23-24 h** | **Pre dose** | **1-2 h** | **5-6 h** | **23-24 h** | **Pre dose** | **1-2 h** | **5-6 h** | **23-24 h** | **Pre dose** | **1-2 h** | **5-6 h** | **23-24 h** |
| **Total no. of rat** | | **6** | **6** | **6** | **6** | **6** | **6** | **6** | **6** | **6** | **6** | **6** | **6** | **6** | **6** | **6** | **6** |
| Posture | Rearing (Normal) | -- | -- | -- | -- | 1 | -- | -- | -- | -- | 1 | 1 | -- | 2 | 1 | -- | 1 |
|  | Asleep, lying on side or curled up (Normal) | 1 | -- | 2 | 1 | 3 | -- | 1 | 1 | 2 | -- | 1 | 1 | -- | -- | 2 | 3 |
|  | Sitting normally, feet tucked in (Normal) | 2 | -- | 1 | -- | 1 | 4 | -- | -- | 3 | 1 | 1 | 1 | 2 | 1 | -- | -- |
|  | Sitting or standing alert, watching (Normal) | 3 | 6 | 3 | 5 | 1 | 2 | 5 | 5 | 1 | 4 | 3 | 4 | 2 | 4 | 4 | 2 |
| Abnormal vocalization | Absent (Normal) | 6 | 6 | 6 | 6 | 6 | 6 | 6 | 6 | 6 | 6 | 6 | 6 | 6 | 6 | 6 | 6 |
| Tremors | Absent (Normal) | 6 | 6 | 6 | 6 | 6 | 6 | 6 | 6 | 6 | 6 | 6 | 6 | 6 | 6 | 6 | 6 |
| Convulsions | Absent (Normal) | 6 | 6 | 6 | 6 | 6 | 6 | 6 | 6 | 6 | 6 | 6 | 6 | 6 | 6 | 6 | 6 |
| Touch escape | No Resistance (Normal) | 6 | 6 | 6 | 6 | 6 | 6 | 6 | 6 | 6 | 6 | 6 | 6 | 6 | 6 | 6 | 6 |
| Reactivity to handling | Squeaks or does not squeak but exhibits mild resistance; easy to handle  (Normal) | 6 | 6 | 6 | 6 | 6 | 6 | 6 | 6 | 6 | 6 | 6 | 6 | 6 | 6 | 6 | 6 |
| Fur appearance | Normal hair coat | 6 | 6 | 6 | 6 | 6 | 6 | 6 | 6 | 6 | 6 | 6 | 6 | 6 | 6 | 6 | 6 |
| Salivation | No wetness around mouth (Normal) | 6 | 6 | 6 | 6 | 6 | 6 | 6 | 6 | 6 | 6 | 6 | 6 | 6 | 6 | 6 | 6 |
| Lacrimation | No Lacrimation (Normal) | 6 | 6 | 6 | 6 | 6 | 6 | 6 | 6 | 6 | 6 | 6 | 6 | 6 | 6 | 6 | 6 |
| Piloerection | Piloerection absent (Normal) | 6 | 6 | 6 | 6 | 6 | 6 | 6 | 6 | 6 | 6 | 6 | 6 | 6 | 6 | 6 | 6 |
| Exophthalmos | Eye ball centrally located (Normal) | 6 | 6 | 6 | 6 | 6 | 6 | 6 | 6 | 6 | 6 | 6 | 6 | 6 | 6 | 6 | 6 |
| Pupil size | Normal | 6 | 6 | 6 | 6 | 6 | 6 | 6 | 6 | 6 | 6 | 6 | 6 | 6 | 6 | 6 | 6 |
| Body/Muscle tone | Normal | 6 | 6 | 6 | 6 | 6 | 6 | 6 | 6 | 6 | 6 | 6 | 6 | 6 | 6 | 6 | 6 |
| Arousal level | Bright, alert and appropriately responsive to the surrounding environment - keeps guard up and engages in  exploratory activity (Normal) | 6 | 6 | 6 | 6 | 6 | 6 | 6 | 6 | 6 | 6 | 6 | 6 | 6 | 6 | 6 | 6 |
| Hypoactivity | Hypo activity absent | 6 | 6 | 6 | 6 | 6 | 6 | 6 | 6 | 6 | 6 | 6 | 6 | 6 | 6 | 6 | 6 |
| Hyperactivity | Hyperactivity absent | 6 | 6 | 6 | 6 | 6 | 6 | 6 | 6 | 6 | 6 | 6 | 6 | 6 | 6 | 6 | 6 |
| Grooming | Absent / Present | 6 | 6 | 6 | 6 | 6 | 6 | 6 | 6 | 6 | 6 | 6 | 6 | 6 | 6 | 6 | 6 |
| Palpebral closure | Eyelids wide open | 6 | 6 | 6 | 6 | 6 | 6 | 6 | 6 | 6 | 6 | 6 | 6 | 6 | 6 | 6 | 6 |
| Tremors | Tremor absent | 6 | 6 | 6 | 6 | 6 | 6 | 6 | 6 | 6 | 6 | 6 | 6 | 6 | 6 | 6 | 6 |
| Twitches | Absent | 6 | 6 | 6 | 6 | 6 | 6 | 6 | 6 | 6 | 6 | 6 | 6 | 6 | 6 | 6 | 6 |
| Clonic convulsions | Absent (Normal) | 6 | 6 | 6 | 6 | 6 | 6 | 6 | 6 | 6 | 6 | 6 | 6 | 6 | 6 | 6 | 6 |
| Tonic convulsions | Absent (Normal) | 6 | 6 | 6 | 6 | 6 | 6 | 6 | 6 | 6 | 6 | 6 | 6 | 6 | 6 | 6 | 6 |
| Ataxia | Ataxia absent | 6 | 6 | 6 | 6 | 6 | 6 | 6 | 6 | 6 | 6 | 6 | 6 | 6 | 6 | 6 | 6 |
| Hypotonia | Hypotonia absent | 6 | 6 | 6 | 6 | 6 | 6 | 6 | 6 | 6 | 6 | 6 | 6 | 6 | 6 | 6 | 6 |
| Gait | Head is horizontal; abdomen rises slightly above floor, limbs moves in a coordinated manner with slight up and down  movement of the body during walking (Normal) | 6 | 6 | 6 | 6 | 6 | 6 | 6 | 6 | 6 | 6 | 6 | 6 | 6 | 6 | 6 | 6 |
| Posture | Animal walks upright, with the back straight and pelvis  off the floor (Normal) | 6 | 6 | 6 | 6 | 6 | 6 | 6 | 6 | 6 | 6 | 6 | 6 | 6 | 6 | 6 | 6 |
| Stereotypy | Absent | 6 | 6 | 6 | 6 | 6 | 6 | 6 | 6 | 6 | 6 | 6 | 6 | 6 | 6 | 6 | 6 |
| Abnormal behavior | Abnormal behavior absent | 6 | 6 | 6 | 6 | 6 | 6 | 6 | 6 | 6 | 6 | 6 | 6 | 6 | 6 | 6 | 6 |
| Breathing | Normal | 6 | 6 | 6 | 6 | 6 | 6 | 6 | 6 | 6 | 6 | 6 | 6 | 6 | 6 | 6 | 6 |
| Defecation | Absent/Present with normal quantity and appearance | 6 | 6 | 6 | 6 | 6 | 6 | 6 | 6 | 6 | 6 | 6 | 6 | 6 | 6 | 6 | 6 |
| Urination | Absent/Present with normal quantity and appearance | 6 | 6 | 6 | 6 | 6 | 6 | 6 | 6 | 6 | 6 | 6 | 6 | 6 | 6 | 6 | 6 |
| Somatosensory/ Touch response | Locomotor orientation/flinch or startle as an evidence of perception (Normal) | 6 | 6 | 6 | 6 | 6 | 6 | 6 | 6 | 6 | 6 | 6 | 6 | 6 | 6 | 6 | 6 |
| Visual approach response | Slowly approaches, sniffs and pulls back/flinch or startle as evidence of perception (Normal) | 6 | 6 | 6 | 6 | 6 | 6 | 6 | 6 | 6 | 6 | 6 | 6 | 6 | 6 | 6 | 6 |
| Pupillary light reflex | Pupil contracts (Normal) | 6 | 6 | 6 | 6 | 6 | 6 | 6 | 6 | 6 | 6 | 6 | 6 | 6 | 6 | 6 | 6 |
| Auditory startle reflex | Mild reaction with twitching of the ears or head indicating that the noise was perceived (Normal) | 6 | 6 | 6 | 6 | 6 | 6 | 6 | 6 | 6 | 6 | 6 | 6 | 6 | 6 | 6 | 6 |
| Palpebral reflex | Eyelid blinks (Normal) | 6 | 6 | 6 | 6 | 6 | 6 | 6 | 6 | 6 | 6 | 6 | 6 | 6 | 6 | 6 | 6 |
| Tail pinch response | Looks back, moves forward  and lightly squeaks (normal) | 6 | 6 | 6 | 6 | 6 | 6 | 6 | 6 | 6 | 6 | 6 | 6 | 6 | 6 | 6 | 6 |
| Righting reflex | Present (Normal) | 6 | 6 | 6 | 6 | 6 | 6 | 6 | 6 | 6 | 6 | 6 | 6 | 6 | 6 | 6 | 6 |

Supplementary Table 2S. Grouping and Allocation of Animals

| Group No. | Treatment | Colour Code | Dose (mg/kg) | Dose Strength (mg/mL) | Dose Volume (mL/kg) | N | Sex | Rats numbers |
| --- | --- | --- | --- | --- | --- | --- | --- | --- |
| G1 | Vehicle control | White | 0 | 0 | 10 | 6 | F | 1-6 |
| G2 | Synacinn™ | Green | 250 | 25 | 10 | 6 | F | 7-12 |
| G3 |  | Blue | 750 | 75 | 10 | 6 | F | 13-18 |
| G4 |  | Orange | 2000 | 200 | 10 | 6 | F | 19-24 |

N: No. of rats; F: Female

Supplementary Table 3S. Parameters of Functional Observation Battery (FOB)

| **Parameters** | **Score/Unit** | **Description** |
| --- | --- | --- |
| Home Cage Observations | | |
| Posture | 0-8 | 0-Sitting or standing alert, watching (Normal), 1-Sitting normally, feet tucked in (Normal), 2-Sitting but head hung down, 3-Flattened, Limbs spread out with abdomen pressed to floor, 4-Asleep, lying on side or curled up (Normal), 5-Rearing (Normal), 6-Circling, 7-Hunched back, back is rounded, even when walking, 8-Head bobbing |
| Abnormal vocalization | 0-1 | 0-Absent (Normal), 1-Present |
| Tremors | 0-1 | 0-Absent (Normal), 1-Present |
| Convulsions | 0-1 | 0-Absent (Normal), 1-Present |
| Hand Held Observations | | |
| Touch escape | 0-2 | 0-No Resistance (Normal), 1-Difficult to remove, 2-Very difficult to remove animal/ animal is aggressive with or without vocalization |
| Reactivity to handling | 0-2 | 0-Squeaks or does not squeak but exhibits mild resistance; easy to handle (Normal), 1-Animal freezes and does not move; becomes rigid in hand (Difficult), 2-Struggles, squirms, appears distressed, attempts to bite (Very difficult) |
| Fur appearance | 0-1 | 0-Normal hair coat, 1-Abnormal hair coat (Stained /Soiled fur etc.) |
| Salivation | 0-2 | 0-No wetness around mouth (Normal), 1-Salivation present (Wetness only around the mouth), 2-Copious salivation |
| Lacrimation | 0-2 | 0-No Lacrimation (Normal), 1-Lacrimation present (Wetness only around the eyes), 2-Tears flow from eyes |
| Piloerection | 0-1 | 0-Piloerection absent (Normal), 1-Piloerection present |
| Exophthalmos | 0-1 | 0-Eye ball centrally located (Normal), 1-Eye ball appears to bulge (Exophthalmia) |
| Pupil size | 0-2 | 0-Normal, 1-Reduced diameter (Miosis), 2-Increased diameter (Mydriasis) |
| Body/muscle Tone | 0-2 | 0-Normal, 1-Reduced tone, 2-Increased tone |
| Observations in Standard Arena | | |
| Arousal level | 0-3 | 0-Bright, alert and appropriately responsive to the surrounding environment - keeps guard up and engages in exploratory activity (Normal), 1-Sometimes does not move but engages in exploratory activity/moves only head (Decreased), 2-Excited or tense; sudden lurches forward and stillness (Increased), 3-Very high (hyper alert, excited) |
| Hypoactivity | 0-3 | 0-Hypoactivity absent, 1-Sometimes does not move but engages in exploratory activity, 2-Mildly numb; only moves head (Sedation), 3-Sleeps except when aroused by strong stimuli (Stupor) |
| Hyperactivity | 0-2 | 0-Hyperactivity absent, 1-Mildly excited or tense; sudden lurches forward and stillness, 2-Highly alert; runs or moves body suddenly |
| Grooming | 0-1 | 0-Absent / Present, 1-Excessive grooming |
| Palpebral closure | 0-2 | 0-Eyelids wide open (Normal), 1-Eyelids partially closed, 2-Eyelids completely closed |
| Tremor | 0-2 | 0-Tremor absent (Normal), 1-Intermittent tremors, 2-Continuous tremors |
| Twitches | 0-2 | 0-Absent (Normal), 1-Present intermittently, 2-Present continuously |
| Clonic convulsions | 0-1 | 0-Absent (Normal), 1-Present |
| Tonic convulsions | 0-1 | 0-Absent (Normal), 1-Present |
| Ataxia | 0-3 | 0-Ataxia absent (Normal), 1-Slight, 2-Considerable without falling, 3-Considerable with frequent falling |
| Hypotonia | 0-3 | 0-Hypotonia absent (Normal), 1-Incomplete extension at tarsus, no impairment of gait, 2-Incomplete extension of tarsus, stifle, and hip; pelvis tipped; impaired gait, drags one limb intermittently, 3-Drags both hindlimbs |
| Gait | 0-1 | 0-Head is horizontal, abdomen rises slightly above floor, limbs moves in a coordinated manner with slight up and down movement of the body during walking (Normal), 1-Abnormal |
| Posture | 0-1 | 0-Animal walks upright, with the back straight and pelvis off the floor (Normal), 1-Abnormal |
| Stereotypy | 0-3 | 0-Absent, 1-Occasionally present, 2-Frequently present, 3-Continuously present |
| Abnormal behaviour | 0-1 | 0-Absent (Normal), 1-Present |
| Breathing | 0-3 | 0-Normal, 1-Rapid and shallow, 2-Slow and deep, 3-Dyspnoea with or without open mouth breathing |
| Defecation | 0-1 | 0-Absent/Present with normal quantity and appearance (Normal), 1-Excessive quantity or abnormal appearance |
| Urination | 0-1 | 0-Absent/Present with normal quantity and appearance (Normal), 1-Excessive quantity or abnormal appearance |
| Rearing | No. of counts | The number of times the animal raised its front feet off the floor was considered rearing |
| Manipulative Tests | | |
| Somatosensory/Touch response | 0-2 | 0-Locomotor orientation/flinch or startle as an evidence of perception (Normal), 1-Freezing or withdrawal, 2-Bizarre response, aggressiveness, vocalization or biting |
| Visual approach response | 0-2 | 0-Slowly approaches, sniffs and pulls back/flinch or startle as evidence of perception (Normal), 1-Freezing or withdrawal, 2-Bizarre response, aggressiveness, vocalization or biting |
| Pupillary light reflex | 0-1 | 0-Pupil contracts (Normal), 1-Pupil does not contract (Fixed pupil) |
| Auditory startle reflex | 0-2 | 0-Mild reaction with twitching of the ears or head indicating that the noise was perceived (Normal), 1-No response, 2-Exaggerated reaction - jumps, attacks |
| Palpebral reflex | 0-1 | 0-Eyelid blinks (Normal), 1-Does not blink |
| Tail pinch response | 0-2 | 0-Looks back, moves forward and lightly squeaks (normal), 1-Reduced reaction, 2-Exhibits a violent overreaction such as jumping, biting, squeaking or attack |
| Righting reflex | 0-1 | 0-Present (Normal), 1-Absent |
| Landing foot splay | mm | A recording paper was spread on a testing platform. The bottom of the animal's hind limbs was pressed against ink pad. Animal was held by neck or back in a horizontal position and dropped when it was in rest, stomach down on the recording paper at approximately 30 cm above the testing platform. The same procedure was performed thrice and the distance between the two heels was recorded. |
| Motor Activity | No. of counts | Motor activity was measured using an automated motor activity measuring system. Activity meter is an infrared photo-cell based lab animal activity and behaviour monitoring system which was used to measure the locomotor activity of animals. Activity was measured for a period of 30 minutes for each animal and time point. |
| Grip Strength | g | At each time point, forelimb and hindlimb grip strength was measured manually using a Grip Strength Meter. The grip strength meter measures the maximal muscle strength of forelimbs and hindlimbs. For each animal, 3 readings were recorded for both forelimb and hindlimb. |
| Body Temperature | °C | Rectal temperature was recorded at the end of FOB observations at each time point using a digital thermometer. |
| Body Weight | g | Body weight was recorded prior to the dose administration and after the 24 h time point observation. |


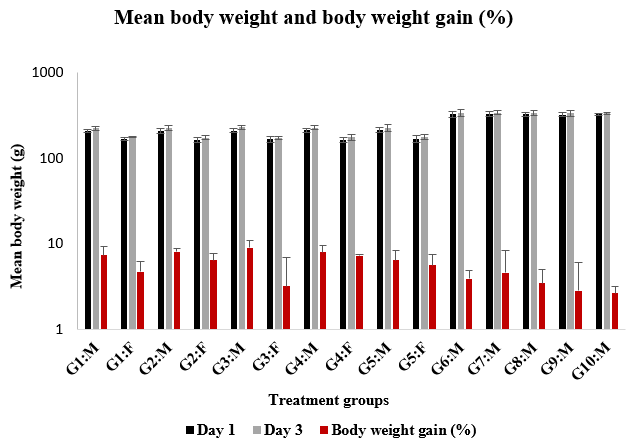


Supplementary Fig. S1. Mean body weight and body weight gain (%). Data represent the Mean±SD. M: Male; F: Female; G1: Vehicle Control: 0 mg/kg/day; G2: Synacinn™: 300 mg/kg/day; G3: Synacinn™: 600 mg/kg/day; G4: Synacinn™: 1000 mg/kg/day; G5: Synacinn™: 2000 mg/kg/day; G6: Vehicle Control: 0 mg/kg/day; G7: Synacinn™: 500 mg/kg/day; G8: Synacinn™: 1000 mg/kg/day; G9: Synacinn™: 2000 mg/kg/day; G10: Cyclophosphamide monohydrate: 25 mg/kg.


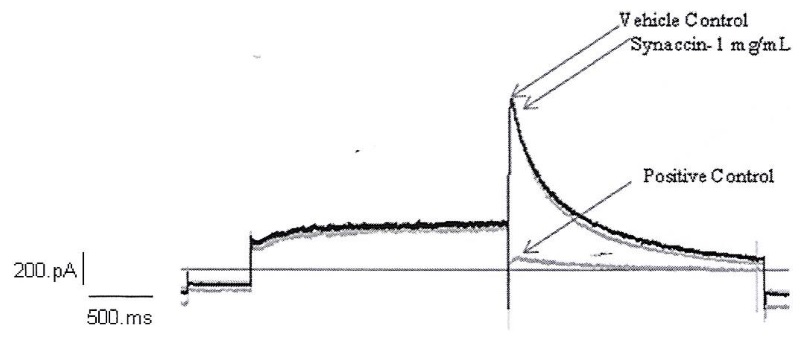
Supplementary Fig. S2 Superimposed records of hERG potassium currents


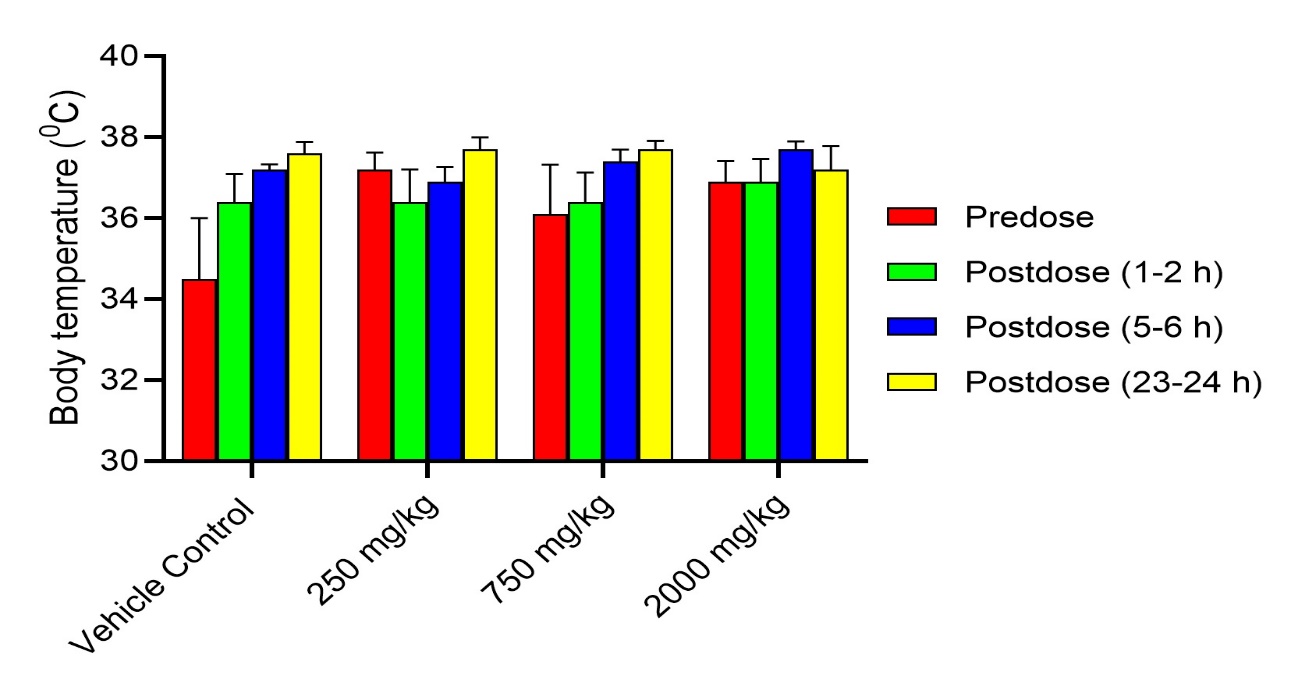


Supplementary Fig. S3 Body temperature of Sprague Dawley rats at predose and postdose. Data represent the mean of respective group measurements and are expressed as Mean±SEM


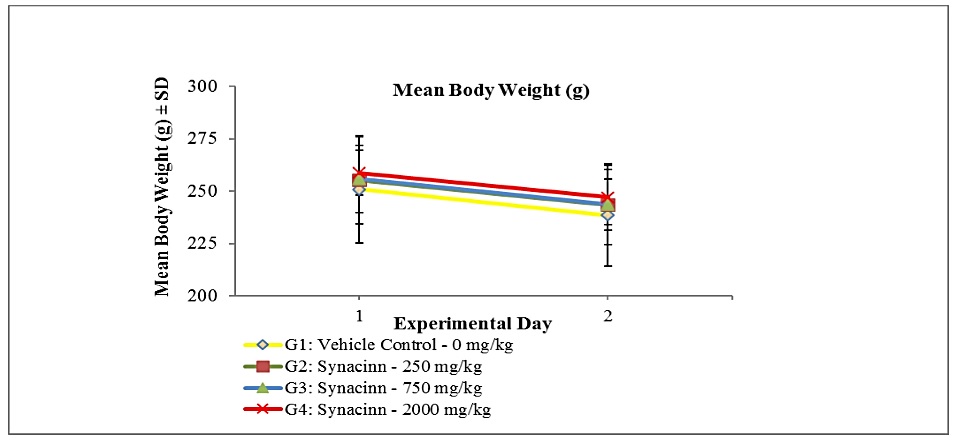


Supplementary Fig. S4 Body weight of Sprague Dawley rats of predose (Day 1) and 24 hour postdose (Day 2)
